# Valorisation of Bovine Sweet Whey and Sunflower Press Cake Blend through Controlled Fermentation as Platform for Innovative Food Materials

**DOI:** 10.3390/foods11101417

**Published:** 2022-05-13

**Authors:** Nicola Mangieri, Davide Ambrosini, Stefano Baroffio, Ileana Vigentini, Roberto Foschino, Ivano De Noni

**Affiliations:** Department of Food, Environmental and Nutritional Sciences, Università degli Studi di Milano, 20122 Milano, Italy; nicola.mangieri@unimi.it (N.M.); dvd.ambrosini@gmail.com (D.A.); stefano.baroffio@studenti.unimi.it (S.B.); ileana.vigentini@unimi.it (I.V.); ivano.denoni@unimi.it (I.D.N.)

**Keywords:** lactic acid bacteria, fermentation, sunflower press cake, sustainability, sweet whey, yeasts

## Abstract

The current environmental challenge is pushing food systems towards more sustainable models of production that require reorganizing of processes by re-using side products still containing nutrients. This work aimed at valorising a mix of bovine sweet whey and sunflower press cake, through targeted fermentation. After preliminary screening based on growth rate, final pH, lactose/galactose assimilation, phytase activity, six Lactic Acid Bacteria strains (*Lacticaseibacillus casei*, *L. paracasei* (2), *Lactococcus lactis*, *Lentilactobacillus parakefiri* and *Leuconostoc pseudomesenteroides*) and three yeasts (*Kluyveromyces lactis*, *K. marxianus* and *Torulaspora delbrueckii*) were co-cultivated in pairs in microcosms (1-part ground press cake: 4-parts whey). All tested microorganisms were able to grow and acidify the blend: the LAB counts increased during the incubation (26 °C for 48 h) of +2.80 log CFU/g, whereas yeasts counts were of +1.98 log CFU/g, with significant differences among the different associations (*p* < 0.01). Mould counts were always <3 log CFU/g. Interestingly, the bacterial contaminants count significantly varied in samples with different pairs of strains (*p* < 0.001). Acidification level, acetic acid and ethanol contents were the limiting factors affecting the growth of spoilage micro-organisms. Best performances were attained in microcosms inoculated with *L. lactis* or *L. paracasei* and *K. lactis* or *K. marxianus*.

## 1. Introduction

In 2019, the European Commission ruled on the European Green Deal, a strategic program of integrated policies that has the goal to make Europe carbon neutral by 2050, transforming the actual economy to a more sustainable system of managing the available resources. In this context, a subset of the program, called The Farm to Fork action, aims at the transition to eco-friendly and healthy food systems for people and planet [1]. For this purpose, the food industry needs to move from a linear to a circular model of production that requires to rethink or rearrange the manufacturing of goods to reduce wastes and losses and to facilitate recovery, as well as recycling of materials. These targets can be reachable with closed-loop production approaches [2] where the building of innovative and sustainable supply chain networks would generate economic convenience for stakeholders by converting food wastes and by-products into ingredients.

Waste, as defined by EU Council, is the material intended to be discarded by the manufacturer and, within this definition, all the side products of food industry can be generally included together with scraps and leftovers [3]. The European research project REFRESH [4], identifies the whey and the press cake from oil seeds as among the top food wastes that deserve innovative solutions for recycling because of the high nutritional properties they retain, overall proteins.

The dairy industry is the second largest component of food waste production worldwide [5]. The EU is the largest producer area of whey with an estimated total amount over 55 million tons, with more than 4 million tons of dry matter production [6]. Large dairy companies mainly transform whey into dried products, such as whey powder, whey protein concentrates, and lactose for being formulated in food and pharmaceutical preparations. Conversely, small, and medium-sized cheese plants cannot afford the equipment to manage this perishable side product; thus, it is generally destined for livestock breeding, partially used for making typical local products (Italian Ricotta, Balkan Urda, Norwegian Mysost) or illegally discarded in the environment [7]. Considering that whey keeps about 55% of the milk solids and that its disposal as wastewater results in heavy pollution with high values of Chemical Oxygen Demand (COD) and Biochemical Oxygen Demand (BOD) parameters, there is a strong demand to give a sustainable solution for using this by-product with an integrated approach involving different food chains [8].

In Europe, sunflower is the third most-used crop for oil seeds production and after the oil extraction approximately 6 million tons of by-products per year are left behind, still having a high content of valuable compounds [9]. Moreover, the sunflower oil production is increasing because of the decision of some bakery and chips companies to reduce or give up the use of the palm oil (https://nuseed.com/eu/global-trends-in-sunflower-production/ (accessed on 12 April 2022)). From a nutritional point of view, press cake is considered edible and frequently used as animal feed [10]. Especially, press cakes from cold pressing as is standard for the production of organic oils, stand out with their high protein content (24% whole, 29% dehulled), fibres (30% whole, 9% dehulled) and polyphenols (4% whole, 1% dehulled) [11].

Fermentation is universally conceived as a sustainable process to enhance sensory and nutritional aspects of raw food materials, being the most ancient, inexpensive, and widespread way in the world to preserve food. Successful manufacturing of a food matter through microbial activities mainly relies on inoculation with selected strains able to early dominate the indigenous populations and improve the product safety and quality by inhibition of pathogenic/spoilage microorganisms and development of flavour components or increase the bio-availability of some nutrients [12]. In this case study, the sweet whey provides the aqueous component of the mixture, a readily available carbon source for microbial growth and a fairly good content of proteins with high nutritional value [13], whereas the sunflower press cake supplies proteins, fibres and lipids with unsaturated fatty acids. Until now, the information on the use of microbial consortia aimed at fermenting mixtures of side products deriving from dairy and oil industries is nil. The objective of the present study was to investigate the behaviour of association formed by Lactic Acid Bacteria (LAB) and potential lactose-fermenting yeast species able to well grow and positively affect the safety and quality of the blend.

A key point to create more sustainable food systems is to find simple processes of transformation for small and medium-sized producers that do not involve change or require big investment in technology. This work is part of an ERA-NET project cofounded by a Joint Call Susfood2 and Core Organic organizations entitled “Towards Sustainable and Organic Food Systems” (https://susfood-db-era.net/main/node/29334 (accessed on 12 April 2022)) pointing to attain secure food supply through a transition from current linear productions to sustainable cyclical-oriented systems. With a targeted Technology Readiness Level of five, this research program addresses the valorisation of side products through fermentation by creating an innovative blended food material (platform) with improved technological and nutritional functionalities, that can be the basis for the development of new products for the human consumption.

## 2. Materials and Methods

### 2.1. Isolation and Identification of Micro-Organisms

The LAB and yeasts were isolated from kefir beverages or supplied by international collection as described in Table 1. The LAB strains were cultivated in de Man-Rogosa-Sharpe (MRS) broth or agar medium (Difco, Sparks, MD, USA); incubation was performed at 30 °C for 48 h. Yeasts strains were grown on Yeast Peptone Dextrose broth or agar medium [14]; incubation was performed at 26 °C for 48 h. Stock cultures for long duration were prepared in the relevant media with added 20% (*v*/*v*) glycerol and stored at −80 °C.

As regards the isolation of micro-organisms from kefir samples, after decimal dilutions in peptoned water (10 g/L peptone, pH 6.4), appropriate aliquots were plated on MRS agar medium supplemented with cicloeximide 100 mg/L for LAB and on Chloramphenicol Glucose Agar (GCA) (Scharlab, Sentemenat, Spain) for yeasts. Single colonies from plates at the highest dilutions were picked and at least two consecutive times streaked to obtain pure cultures.

Species identification of the isolates was carried out by DNA sequence analysis, targeting the 16S rDNA gene and D1/D2 domain of the 26S rDNA gene for LAB and yeast strains, respectively. The DNA from the LAB was extracted by using phenol/chloroform protocol according to Green and Sambrook [15]. Universal primers for bacteria named BSF8 (5′-AGAGTTTGATCCTGGCTCAG-3′) and BSR1541 (5′-AAGGAGGTGATCCAGCCGCA-3′) [16] were used and the relevant amplification conditions were those reported by Vigentini et al. [17]. The DNA extraction from yeasts was performed according to Querol et al. [14]. Amplification was carried out using primers NL1 (5′-GCATATCAATAAGCGGAGGAAAAG-3′) and NL4 (5′-GGTCCGTGTTTCAAGACGG-3′), as previously described by Kurtzman and Robnett [18]. The PCR products were purified with the EuroClone^®^ spinNAker purification kit (Pero, Milano, Italia) and then sequenced by external provider (Eurofins Genomics, Vimodrone, Italy). The obtained sequences were compared by Basic Local Alignment Search Tool (BLAST) (http://www.ebi.ac.uk/blastall/nucleotide.html (accessed from 30 August 2021)) and species identification wa attained (homology > 97%).

### 2.2. Strain Discrimination by Molecular Fingerprinting

Isolates ascribed to the same species were subjected to fingerprinting techniques to discriminate them at the strain level. For LAB strains, genomic DNA samples were amplified by Randomly Amplified Polymorphic PCR (RAPD-PCR) with primer M13 according to Rosetti and Giraffa [19] and by Repetitive Extragenic Palindromic sequence-based PCR (REP-PCR) with primer BOX-A1 (5′-CATACGGCAAGGCGACGCT-3′) as suggested by Freitas et al. [20]. As regards the yeast strains, the enzyme restriction analysis of mitochondrial DNA was performed as previously described by Cordero-Bueso et al. [21] using *Hinf*I (ThermoFisher Scientific, Waltham, MA, USA).

### 2.3. Determination of Phytase Activity and Carbohydrates Assimilation Profile of the Strains

The LAB strains were investigated for their ability to use phytic acid as phosphorous source through a modified MRS broth (MRS-MOPS), in which inorganic phosphate (KH_2_PO_4_) was substituted by 0.65 g/L of phytic acid sodium salt hydrate (Sigma-Aldrich, St. Louis, MO, USA) and 0.1 M3-[N-Morpholino] propane-sulfonic acid (MOPS, Sigma-Aldrich, St. Louis, MO, USA). The bacteria were grown on MRS-MOPS medium in two consecutive times of 24 h at 30 °C with an inoculum of 5% (*v*/*v*) [22]. Likewise, yeasts were cultivated in minimum media containing only glucose (10 g/L) and 0.65 g/L of phytic acid sodium salt hydrate with the same protocol described for LAB with the incubation at 26 °C.

Cells were separated by centrifugation (8515× *g* for 15 min at 4 °C) and 250 μL of supernatant were added with 250 μL of 2 mM phytic acid prepared in 100 mM sodium acetate–acetic acid buffer (pH 5.5). Then samples were incubated at 50 °C for 15 min. The reaction was stopped by adding 500 μL of 10% (*w*/*v*) trichloroacetic acid solution (Merck, Darmstadt, Germany). The inorganic phosphorus released was quantified by the ammonium molybdenum method [23]. One unit of phytase activity (U) was defined as the amount of enzyme that produces 1 μmol of inorganic phosphorus per minute at 50 °C.

Yeasts strains were evaluated in terms of carbohydrates assimilation by API^®^ ID 32 C kit by BioMérieux™ (Marcy-l’Étoile, Francia) according to the supplier’s recommendation. As regards the LAB strains, 350 μL of tryptone (WWR, Leuven, Belgium) solution (200 g/L) were added to each ampoule of API C Medium before inoculation of the pure culture. Strips were incubated in anaerobiosis at 30 °C for a week.

### 2.4. Growth Test in Bovine Sweet Whey

A first series of growth trials was carried out in commercial bovine sweet whey powdered (Flowhey, Lactalis ingredients, Bourgbarre, France), reconstituted at 6% (*w*/*v*) with demineralized water and heat treated at 100 °C (microwave oven ZM30TH, Zanussi, Pordenone, Italy) for 1 min. Small-scale growth experiments were performed in sterile Greiner Bio-One™ 24-wells plates (Greiner, Kremsmünster, Austria) with a flat bottom. Wells were filled with 1 mL of whey, inoculated with an appropriate volume of fresh cultures of one bacterial strain and one yeast strain to obtain a starting cell concentration of 10^6^ CFU/mL and 10^5^ CFU/mL, respectively.

To investigate the microbial pairs with the best performance in growth rate and acidification, each bacterial strain was co-inoculated with each yeast strain. The culture of each single strain, at the same growth conditions, were used as control tests. The plates were incubated at 26 °C for 48 h and the optical density (OD) at 600 nm was monitored every 30 min by using BioTek^®^ Eon microplate reader (Winooski, VT, USA). At the starting (t_0h_) and the end time (t_48h_), the pH values were measured (Jenway™ 3510 pH meter, Cole-Parmer, Stone, UK). At the end of incubation, 10 µL aliquot from each well were microscopically observed at 600× (Standard 25, Zeiss, Oberkochen, Germany) to verify potential contaminations. Each experiment was conducted in triplicate. The absorbance values were analysed using R Core Team (R Core Team, 2017) software package “Growthcurver” to fit the experimental data with a logistic equation for extrapolating maximum growth rate.

### 2.5. Fermentation Trials in Microcosms

A second series of co-cultivation experiments were performed in a 50 mL sterile Falcon^®^ tube containing 32 mL of reconstituted whey and 8 g of whole sunflower press cake (Ölmühle Moog, Lommatzsch, Germany) obtained by cold pressing and milling. Information on the press cake composition is given in Table S1 (Supplementary Materials). Fresh cultures of the strains selected in the previous screening step were prepared and relevant appropriate volumes were added to the tube to obtain 10^6^ CFU/mL for LAB and 10^5^ CFU/mL for yeasts, as starting cell concentrations. Each microcosm, containing one bacterial and one yeast strain, was stirred with a vortex for 5 min and then incubated at 26 °C for 48 h in an orbital shaker (Stuart Gyro-Rocker STR9, Cole-Parmer, Stone, UK) at 100 rpm. The image of a microcosm is shown in Supplementary Materials (Figure S1). At t_0h_ and t_48h_, microcosms were microbiologically analysed by plate count technique using different growth media: MRS agar plus cicloeximide for LAB enumeration with incubation at 30 °C for 48 h, Chloramphenicol Glucose Agar (GCA) for yeasts and moulds enumeration with incubation of 26 °C for 72 h, sugar-free agar for mesophilic bacterial contaminants with incubation at 30 °C for 48 h (Scharlab, Sentemenat, Spain). Also, the pH value was measured at t_0h_ and t_48h_ of the fermentation time. Each experiment was replicated in three independent tests.

### 2.6. Determination of Sugars and Organic Acids by HPLC Method

Sugars and organic acids from microcosms samples were determined at t_0h_ and t_48h_. Both analyses were carried out using an HPLC Alliance 2695 pump system (Waters, Milford, MA, USA) equipped with a model 2414 differential refractometer (Waters, Milford, MA, USA). The HPLC separation of organic acids was conducted using an Aminex HPX-87H column (300 mm i.d. × 7.8 mm) from Bio-Rad (Segrate, Italy) kept at 50 °C. The analytical conditions were as follows: flow 0.6 mL/min, eluent 0.01 N H_2_SO_4_, injection volume 2 µL. The samples were centrifuged at 14,000× *g* for 20 min and then at 16,100× *g* for 10 min. Subsequently, 0.5 mL of supernatant was mixed with 2 mL of MilliQ water (Millipore, Darmstadt, Germany) and then were filtered through a 0.22 μm pore size membrane filter (Millipore, Darmstadt, Germany) before injection. The analysis of sugars was carried out using the same HPLC system and detector, but with two Aminex HPX-87P columns in a series (300 mm i.d. × 7.8 mm, Bio-Rad, Segrate, Italy) kept at 75 °C. The analytical conditions were as follows: flow 0.6 mL/min, eluent MilliQ water, injection volume 5 µL. The samples were centrifuged at 14,000× *g* for 20 min and then at 16,100× *g* for 10 min. Subsequently, 0.75 mL of supernatant was mixed with 3 mL of Biggs reactive and then brought to volume in a 25 mL flask with milliQ water. Finally, the samples were filtered through a 0.22 μm pore size membrane filter (Millipore, Darmstadt, Germany) before injection.

Quantification of organic acids and sugars was performed using aqueous solutions of ethanol and lactic, acetic acids, and galactose, glucose, lactose, sucrose and raffinose as external standards. All sugars and organic acids were of analytical grade (Sigma-Aldrich, St. Louis, MO, USA). Analyses were performed in triplicate.

### 2.7. Statistical Analysis

The ANOVA of the data was elaborated with Statgraphics Centurion software (v. 18, Statistical Graphics Corp., Herndon, VA, USA); the Tukey’s HSD test was used to compare the sample means to evaluate significant differences among mean values of main factors.

## 3. Results

### 3.1. Micro-Organism Identification and Strain Typing

Dominant microorganisms isolated from industrial and homemade kefir samples were successfully identified according to the above-mentioned protocols and the results are reported in Table 1.

To discriminate among LAB strains belonging to the same species, different PCR fingerprinting techniques were used, considering as diverse the isolates that showed different banding patterns. Apart from of *Lactobacillus kefiranofaciens* KMSG-22B (B5) and KMSG-21B isolates, picked up from the same sample, and *Lactococcus lactis* 2KB-1B (B10) and 4BK-11B isolates coming from two commercial kefirs, the remaining strains proved to be different (data not shown). Only one of the isolates revealing the same amplification profile was kept for the further stages.

As regards the yeasts, strain fingerprinting was performed by enzymatic restriction of mitochondrial DNA. Although *Pichia fermentans* 2KB-1Y, 4BK-1Y (L6) and 4BK-2Y (not shown) isolates showed different morphologies on GCA plates, they interestingly pointed out identical restriction profiles confirming that there was probably a unique strain collected from samples which derived from different brands, as previously described for LAB strains, isolated in those same specimens of retail kefir. Only one of these isolates was selected for the next steps. All other yeast strains were identified as different (data not shown).

### 3.2. Phenotypic Characterization of Strains

The results obtained by phytase activity test exhibited considerable differences between the LAB, with a minimum value of 6.6 U for *Leuconostoc pseudomesenteroides* B14 strain and a maximum value of 23.4 U for *Lacticaseibacillus paracasei* B8 strain; the median value was 18.0 U. Conversely, yeasts displayed a very similar behaviour with a mean value of 7.4 U, ranging from 6.6 to 7.8 U (Table 1). The potential ability to degrade phytic acid was considered as criterion to select strains, so only those that exceeded the median value passed to the next step of evaluation.

As regards the carbohydrate assimilation, only sugars that were found in meaningful quantities in the blend were commented on. Nevertheless, the complete patterns of carbohydrates assimilation for all tested strains are reported in Table S2 (Supplementary Materials). In the case of LAB, all strains consumed galactose but *L. pseudomesenteroides* B14, whereas *L. paracasei* B3, *Lactobacillus parakefiri* B6 and *L. kefiri* B7 were unable to grow with only glucose. The strains *L. paracasei* B3 and B8, *L. casei* B4, *L. parakefiri* B6, *L. kefiri* B7 and *L. pseudomesenteroides* B14 did not use lactose. Conversely, *L. paracasei* B1 and *L. lactis* B10, B11 and B12 strains grew with raffinose as unique carbon source.

All the yeast strains were able to use glucose. Galactose, lactose and raffinose were consumed by *Debaryomyces hansenii* L1, *Kluyveromyces lactis* L2 and L9 strains, *Kluyveromyces marxianus* L3, L4 and L7 strains. *Torulaspora delbrueckii* L5 consumed galactose and raffinose. Lactate was used by all the above-mentioned yeasts and the *P. fermentans* L8 strain.

### 3.3. Growth Test in Bovine Sweet Whey

Eleven yeast strains and fourteen bacterial strains were co-cultivated in reconstituted whey by mixing them one by one or alone, for a total of 537 growth trials considering that each experiment was replicated three times. Use of a logistic function for modelling microbial growth, the continuous monitoring of OD at 25 °C for 48 h allowed to estimate the maximum growth rate for each pair. All growth curves are reported in Figure S2 (Supplementary Materials). The mean values and related standard deviation of maximum growth rates obtained from the elaboration of absorbance values in co-cultivation experiments are shown in Table 2. Obviously, the measured OD values over time were the sum of the absorbance of yeast cells plus that of bacterial cells, being not possible to distinguish the contribution of the two populations. Nevertheless, the possibility of reliably modelling the growth curve also for microbial associations has recently been demonstrated by Altilia et al. [24]. The final microscopic observations of each sample allowed to check possible contaminations and confirmed the presence of both types of microorganisms in co-cultures. Data were subjected to one-way ANOVA to assess the effect of the LAB strain and that of the yeast one on the growth kinetics of the 154 tested microbial pairs. As regards the “bacterial strain” factor, the average maximum growth rates of yeast associations inoculated with all *L. lactis* strains were significantly higher (*p* < 0.001) than those inoculated with the remaining LAB strains. Beyond this species, *L. pseudomesenteroides* B14 and *L. paracasei* B8 strains exhibited the best performances in terms of speed of cell development together with the examined yeasts. The median value of maximum growth rate observed for LAB was 0.38 (1/h). Concerning the effect of “yeast strain”, the LAB co-cultivated with the *K. marxianus* L3 and L4, or *T. delbruecki* L5 strains showed significantly higher maximum growth rates (*p* < 0.001) respect to those associations inoculated with the *D. hansenii* L1 strain. The median value of maximum growth rate observed for yeasts was 0.44 (1/h). Only LAB and yeast strains showing values exceeding the relevant median values of maximum growth rate were considered for the subsequent selection.

The mean values and related standard deviation of pH measured at the end of incubation for each pair strains are reported in Table 3. As regards the “bacterial strain” factor, the average final pH reached by yeast associations inoculated with *L. lactis* B10, B11, B12 or *L. pseudomesenteroides* B14, were significantly lower (*p* < 0.001) than those inoculated with the remaining LAB strains. The median pH value detected at the end of incubation for LAB was 4.65. Concerning the effect of “yeast strain”, the bacterial strains co-cultivated with the *K. lactis* L2 and L9, or *K. marxianus* L7, or *Saccharomyces cerevisiae* L11 strains revealed significantly lower final pH (*p* < 0.001) respect to those associations inoculated with the *D. hansenii* L1, or *P. fermentans* L8 and L6, or *Pichia kluyveri* L10 strains. The pH 4.51 was the median value measured for yeasts. Likewise, only LAB and yeast strains showing an endpoint pH value lower than the respective median ones were selected for the next experimental stage.

### 3.4. Fermentation Trials in Microcosms

Starting from results obtained in the previous screening, six LAB strains (*L. paracasei* B2 and B8, *L. casei* B4, *L. parakefiri* B6, *L. lactis* B12 and *L. pseudomesenteroides* B14), and three yeast strains (*K. lactis* L2, *T. delbrueckii* L5 and *K. marxianus* L7) were selected for co-cultivation experiments in a blend material constituted of 80% liquid whey and 20% solid press cake powder.

The microbiological features of the blend samples before the inoculation showed the following mean counts (log CFU/g ± standard deviation): mesophilic bacterial contaminants, 4.07 ± 0.30; moulds, 3.34 ± 0.50; yeasts < 2. Most of the bacterial contaminants were recognized as spore formers through microscopic observations of randomly picked colonies. At the starting time, the mean pH value of the microcosms was 5.97 ± 0.03.

Plate counts were carried out to assess the growth trends of the different microbial groups during the co-cultivation experiments. Each LAB inoculated with each yeast strain proved to be able to grow, overcoming a possible inhibition caused by the high level of polyphenols contained in the press cake component. Starting and final Log CFU/g counts ± standard deviation of investigated microbiological groups are reported in Supplementary Materials (Table S3).

**Figure 1:**
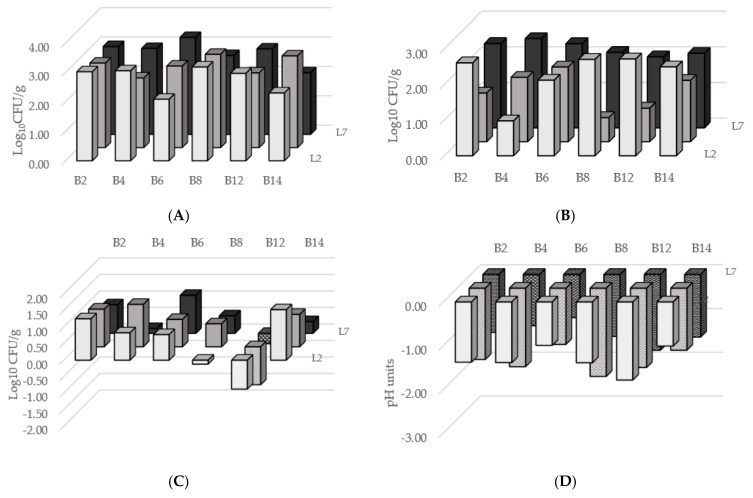
Change of LAB (**A**), yeasts (**B**) and bacterial contaminants (**C**) as difference in log CFU/g plate counts and change of pH (**D**) during the fermentation in microcosms inoculated with different pairs of LAB and yeast strains.

Figure 1A shows the increase (log CFU/g) of LAB cell concentrations without significant differences among the couples (*p* = 0.637): independently from the paired yeast, the lowest development was observed in co-cultures with the *L. pseudomesenteroides* B14 strain, which exhibited an averaged raise of +2.51 log CFU/g. On the other hand, the highest growth was found for associations with the *L. paracasei* B8 strain, which advanced of +3.02 log CFU/g. Among all LAB the mean cell increase in microcosms was of +2.80 log CFU/g.

Regardless of the paired LAB strain, the least increase of yeast population was shown by the microbial pairs with the *T. delbrueckii* L5 strain, which augmented +1.44 log CFU/g on average. Conversely, the highest leap was observed in co-cultures with the *K. lactis* L2 strain that exhibited a significantly (*p* < 0.010) mean rise of +2.27 log CFU/g. The averaged surge in cell concentrations among yeast strains was of +1.98 log CFU/g.

The trend of bacterial contaminants significantly (*p* < 0.001) varied with the different microcosms, even highlighting a decrease in cell concentration in four out of 18 cases (Figure 1C). Without considering the paired yeasts, the microbial associations with *L. lactis* B12 led to a reduction of bacterial contaminants of −0.78 log CFU/g after 48 h of incubation; particularly, when cultivated with the *T. delbrueckii* L5 strain, they diminished of −1.15 log CFU/g. Instead, microcosms with the *L. paracasei* B2 strain showed the highest rise in bacterial contaminant counts (+1.09 log CFU/g), although the worst result occurred in co-cultivation of *L. pseudomesenteroides* B14 and *K. lactis* L2 strains (+1.53 log CFU/g). Regardless of the paired LAB, the couples with the *K. marxianus* L7 strain exhibited the most limited development of bacterial contaminants, which revealed an average augmentation of +0.46 log CFU/g, though there were not significant differences with other yeasts (*p* = 0.883). Interestingly, moulds counts were <3 log CFU/g in all examined samples.

Lowering of the pH value was always observed (Figure 1D), demonstrating the capability of the tested strains to ferment the blend. However, the various microbial combinations highlighted the ability of some pairs of strains to better acidify, as revealed by the significant differences (*p* < 0.001) observe for the decrease in pH value. Independently from the paired yeast, the strongest reduction of pH value was detected in co-cultures with *L. lactis* B12, which displayed a mean drop of −1.78 units. On the other hand, the weakest change was found in co-cultivations with the *L. parakefiri* B6 strain, −1.09 units on average. Without considering the associated bacterial strains, the microcosms inoculated with *T. delbrueckii* L5 reached a lower pH value respect to the other inoculated yeasts (*p* = 0.003), with a mean decrease of −1.66 units.

The chemical analysis of the blend samples before the inoculation of the strains showed the following results expressed as g/100 g ± standard deviation: lactose, 3.47 ± 0.22; glucose, 0.16 ± 0.02; galactose, 0.15 ± 0.02; lactic acid 0.04 ± 0.00. Ethanol and acetic acid were not revealed.

**Figure 2:**
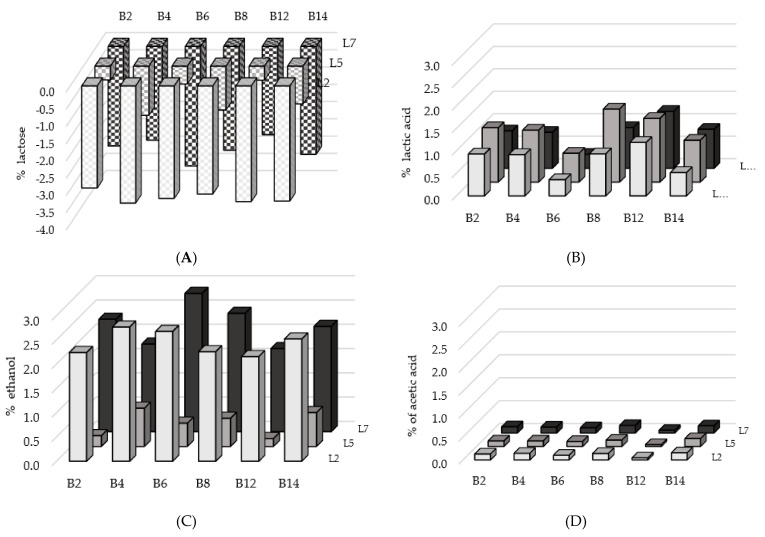
Reduction of lactose content (**A**), production of lactic acid (**B**), ethanol (**C**) and acetic acid (**D**) during the fermentation trials in microcosms inoculated with different pairs of LAB and yeast strains.

The highest consumption of lactose was found in microcosms with the *L. casei* B4 and *L. pseudomesenteroides* B14 strains, with average values greater than 2.50 g/100 g (Figure 2A), while a lower diminution was observed with *L. paracasei* B2 (2.08 g/100 g) and *L. lactis* B12 (2.11 g/100 g) without any significant differences (*p* = 0.957). As expected, the depletion of lactose among the microbial associations inoculated with *K. lactis* L2, *K. marxianus* L7 and *T. delbrueckii* L5 strain was significantly different (*p* < 0.001); without taking in account the paired LAB, the level of decrease for this sugar was on average of −3.24, −2.96 and −0.85 g/100 g, respectively. Actually, *T. delbrueckii* is not a species able to ferment lactose.

The lactic acid content at the end of the incubation resulted from production by LAB and consumption by yeasts and *L. lactis* B12, which can be considered negligible as the samples were in partially anaerobic conditions. The highest concentrations of lactic acid were detected in microcosms inoculated with the *L. lactis* B12 and *L. paracasei* B8 strains, 1.29 and 1.16 g/100 g on average, respectively (Figure 2B). Contrarily, the lowest concentrations (0.44 g/100 g) were revealed in samples with the *L. parakefiri* B6 strain (*p* < 0.001). Regardless of the paired bacteria, microbial associations with the *T. delbrueckii* L5 strain showed an amount of lactic acid, on average 1.16 g/100 g, greater than those co-cultivated with the other two yeasts (*p* = 0.009).

Concerning the formation of ethanol, the samples inoculated with the *L. parakefiri* B6 strain evinced a higher mean content (2.00 g/100 g) than those coupled with the *L. lactis* B12 (1.34 g/100 g), but without significant differences (*p* = 0.861) (Figure 2C). The influence of the yeast strain was clearly evident (*p* < 0.001) as in microcosms with *K. lactis* L2 and *K. marxianus* L7 the average alcohol concentration was 2.21 and 2.43 g/100 g, respectively, whereas in the ones co-cultivated with *T*. *delbrueckii* L5 a mean value of 0.49 g/100 g was observed.

Independently from the associated yeast, the acetic acid production proved to be low (Figure 2D), from 0.05 g/100 g for microbial associations with *L. lactis* B12 to 0.16 g/100 g for those coupled with *L. pseudomesenteroides* B14 (*p* < 0.001). The microcosms inoculated with the *T. delbrueckii* L5 showed a lower quantity of acetic acid (0.11 g/100 g) than those paired with the *K. lactis* L2 strain (0.13 g/100 g), with no significant differences (*p* = 0.742).

## 4. Discussion

Fermentation has been proposed as a strategic process to guarantee the safety and gain potential improvement in nutritional properties [25,26] and it was applied in this work for a blend prepared by mixing bovine sweet whey and ground sunflower press cake. Some critical issues linked to legislative aspects must be considered for ensuring the suitability of the used materials and, on the other hand, for meeting the acceptability of consumers who must be made aware of their choices. Side products can retain residual microbial contamination depending on the hygienic conditions and processes they previously underwent. Because of the unpredictability of this contamination, controlled fermentation with selected strains was adopted to steer the transformation of the mixed materials. The choice of micro-organisms to be used as inoculum was directed towards microbial consortia frequently found in traditional kefir. This approach was driven by the following considerations: (i) the main available carbon source was lactose, so strains should be able to ferment it; (ii) their metabolism should allow a fast lowering of the pH and ethanol production to limit or stop the growth of pathogens and spoilage microbiota; (iii) finally, kefir microorganisms are normally recognized as safe [27,28].

The primary selection of the paired strains was based on the maximum growth rate and pH end point values observed with the associations of LAB and yeasts in reconstituted whey, as well as lactose and/or galactose assimilation and phytase activity of the single strain determined under controlled conditions. This led to the next preparation of 18 different microcosms in which each of the previously selected six LAB were combined with three yeast strains. The experiments, carried out in a mixture composed by four parts of liquid bovine sweet whey and one part of solid press cake, showed that all tested micro-organisms were able to well ferment the blend. The best performance in terms of bacterial inhibition were displayed by couples in which *L. lactis* B12 or *L. paracasei* B8 strains were cultured, both showing a remarkable increase of cell concentration and acidifying capacity; furthermore, the former revealed the highest growth rate among LAB strains and the latter the greatest phytase activity. As regards the yeasts, the microbial associations co-cultivated with the *K. lactis* L2 or *K. marxianus* L7 strains proved, on average, to better restrain the growth of bacterial contaminants, probably due to their capability to quick consume lactose and produce ethanol in the tested conditions. They also showed the highest rise in cell counts during the incubation time. The co-inoculation of these above-mentioned strains allowed to reach pH values below 4.4, which is a recommended safety limit for the growth of bacterial pathogens, and it is definitively proposed for the fermentation of whey and sunflower press cake blend. Notably, despite the initial fungal contamination in the samples, the production of carbon dioxide, ethanol, and acetic acid during the incubation of microcosms resulted in a general hindering of mould growth regardless of the tested microbial associations. On the other hand, significant differences were noted in the behaviour of the diverse tested combinations of micro-organisms towards the contaminating bacteria.

Furthermore, the results pointed out the ability of LAB and yeasts to grow at high content of polyphenols (0.58–0.84 g/100 g, calculated from Weisz et al. [11]). Actually, depending on their structure and concentration, phenolic compounds may exhibit anti-microbial activity because they cause changes in metabolism and impair the cell membrane continuity [29,30]. Nonetheless, Fritsch et al. [31] highlighted a degradation ability of chlorogenic acid of four different LAB suggesting a feasible way to reduce phenolic contents in sunflower meal. Even if we did not know how much polyphenols affected the growth of single inoculated strain, it was evident that a fermentation always took place and, to the best of our knowledge, this is the first time for the examined microbial species.

## 5. Conclusions

Fermentation still has great importance for small-scale industries and household production [32]. Through the transformation of the blend carried out by LAB and yeasts, the project aimed at valorising these two major side products of the European food industry to create a food platform on which new ingredients or finished products can be developed like beverages, semi-solid spreads and snacks, as foreseen in the ERA-NET program within which this work was carried out (https://susfood-db-era.net/main/FERBLEND (accessed on 12 April 2022)). In addition, it could match the market demand of sustainable foods and build new entry points for by-products in food formulations. The results of the present study can serve as a proof of concept for exploiting food resources still considered as waste to increase resource efficiency of the processes and support the circularity of the involved supply chains. This preliminary outcome provides a potential technological solution that might be appealing to various professionals, like food researchers and product developers, to design new functional ingredients or novel foods [33].

## Figures and Tables

**Table 1:** List of the microbial strains used in this work and relative phytase activity.

Code	Origin	Collection Number	Species	Identification (%)	U ^1^
B1	Homemade sugary kefir	LAC CAS 34	*Lacticaseibacillus paracasei*	99.6	17.1
B2	Homemade sugary kefir	LAC YAM 2	*Lacticaseibacillus paracasei*	99.8	20.6
B3	Homemade sugary kefir	DTB-1B	*Lacticaseibacillus paracasei*	99.9	14.8
B4	Homemade sugary kefir	LAC CAS 32	*Lactobacillus casei*	99.6	18.0
B5	Homemade milk kefir	KMSG-22B	*Lactobacillus kefiranofaciens*	97.1	19.7
B6	Homemade milk kefir	KMSG-1B	*Lentilactobacillus parakefiri*	100.0	16.6
B7	Homemade milk kefir	KMSS-1B	*Lentilactobacillus kefiri*	100.0	12.8
B8	Homemade milk kefir	DGL-1B	*Lacticaseibacillus paracasei*	100.0	23.4
B9	Homemade milk kefir	DGL-2B	*Lacticaseibacillus paracasei*	100.0	18.4
B10	Commercial milk kefir	2KB-1B	*Lactococcus lactis*	99.7	20.8
B11	Commercial milk kefir	3KS-1B	*Lactococcus lactis*	100	12.8
B12	Commercial milk kefir	4BK-12B	*Lactococcus lactis*	100.0	10.2
B13	Homemade sugary kefir	LAC FRU 1	*Leuconostoc citreum*	99.5	20.3
B14	Commercial milk kefir	1KP-1B	*Leuconostoc pseudomesenteroides*	99.4	6.6
L1	Commercial milk kefir	2KB-12Y	*Debaryomyces hansenii*	99.6	7.4
L2	Household milk kefir	AVY	*Kluyveromyces lactis*	98.3	7.3
L3	Household milk kefir	CAND 22	*Kluyveromyces marxianus*	100.0	7.4
L4	CBS collection (milk kefir)	CBS 834	*Kluyveromyces marxianus*	100.0	7.5
L5	Commercial milk kefir	1KP-1Y	*Torulaspora delbruecki*	99.6	6.6
L6	Commercial milk kefir	4BK-1Y	*Pichia fermentans*	100.0	7.8
L7	CBS collection (yoghurt)	CBS 397	*Kluyveromyces marxianus*	99.2	7.2
L8	Commercial milk kefir	3KS-1Y	*Pichia fermentans*	99.6	7.8
L9	CBS collection (creamery)	CBS 2359	*Kluyveromyces lactis*	99.8	7.2
L10	Household sugary kefir	DTB-1Y	*Pichia kluyveri*	99.6	7.4
L11	Household milk kefir	DGL-1Y	*Saccharomyces cerevisiae*	99.0	7.3

^1^ One unit of phytase activity (U) was defined as the amount of enzyme that produces 1 μmol of inorganic phosphorus per minute at 50 °C.

**Table 2:** Mean of maximum growth rate values (1/h) ± standard deviation observed in co-cultivation with each LAB strain (from B1 to B14) crossed with each yeast strain (from L1 to L11) in reconstituted bovine sweet whey.

	L1	L2	L3	L4	L5	L6	L7	L8	L9	L10	L11	Average
B1	0.24 ± 0.10	0.29 ± 0.10	0.76 ± 0.53	0.35 ± 0.06	0.33 ± 0.14	0.28 ± 0.24	0.52 ± 0.28	0.16 ± 0.12	0.53 ± 0.48	0.36 ± 0.24	0.35 ± 0.41	0.36 ^abc^ ± 0.30
B2	0.21 ± 0.04	0.36 ± 0.18	0.68 ± 0.29	0.60 ± 0.29	0.27 ± 0.15	0.62 ± 0.41	0.44 ± 0.04	0.20 ± 0.14	0.64 ± 0.63	0.03 ± 0.01	0.38 ± 0.13	0.39 ^abc^ ± 0.32
B3	0.22 ± 0.13	0.33 ± 0.05	0.36 ± 0.09	0.54 ± 0.30	0.33 ± 0.09	0.08 ± 0.08	0.40 ± 0.03	0.16 ± 0.07	0.53 ± 0.27	0.25 ± 0.21	0.61 ± 0.01	0.33 ^ab^ ± 0.21
B4	0.14 ± 0.06	0.32 ± 0.20	0.33 ± 0.07	0.43 ± 0.11	0.29 ± 0.05	0.09 ± 0.03	0.53 ± 0.11	0.21 ± 0.13	0.40 ± 0.21	0.19 ± 0.16	0.67 ± 0.29	0.34 ^ab^ ± 0.21
B5	0.19 ± 0.13	0.30 ± 0.06	0.39 ± 0.09	0.33 ± 0.09	0.38 ± 0.12	0.42 ± 0.35	0.33 ± 0.06	0.51 ± 0.36	0.32 ± 0.10	0.11 ± 0.11	0.57 ± 0.47	0.34 ^ab^ ± 0.21
B6	0.12 ± 0.05	0.42 ± 0.15	0.48 ± 0.21	0.33 ± 0.09	0.40 ± 0.18	0.33 ± 0.06	0.68 ± 0.55	0.27 ± 0.19	0.98 ± 0.22	0.14 ± 0.06	0.31 ± 0.06	0.40 ^abc^ ± 0.30
B7	0.10 ± 0.04	0.32 ± 0.04	0.35 ± 0.06	0.39 ± 0.10	0.23 ± 0.06	0.21 ± 0.18	0.32 ± 0.06	0.12 ± 0.09	0.41 ± 0.20	0.20 ± 0.08	0.45 ± 0.12	0.27 ^a^ ± 0.14
B8	0.59 ± 0.50	0.31 ± 0.04	0.51 ± 0.29	0.65 ± 0.41	0.66 ± 0.18	0.29 ± 0.24	0.78 ± 0.67	0.37 ± 0.15	0.36 ± 0.09	0.33 ± 0.06	0.19 ± 0.04	0.45 ^abcd^ ± 0.31
B9	0.46 ± 0.32	0.33 ± 0.05	0.57 ± 0.26	0.85 ± 0.59	0.55 ± 0.42	0.19 ± 0.14	0.35 ± 0.05	0.04 ± 0.01	0.30 ± 0.03	0.12 ± 0.03	0.10 ± 0.04	0.34 ^ab^ ± 0.32
B10	0.58 ± 0.10	0.84 ± 0.41	0.68 ± 0.23	0.77 ± 0.35	0.67 ± 0.08	0.62 ± 0.49	0.65 ± 0.32	0.49 ± 0.13	0.34 ± 0.11	0.64 ± 0.16	0.74 ± 0.17	0.64 ^d^ ± 0.26
B11	0.62 ± 0.01	0.66 ± 0.26	0.63 ± 0.18	0.41 ± 0.17	0.71 ± 0.06	0.52 ± 0.23	0.62 ± 0.07	0.35 ± 0.03	1.00 ± 0.64	0.60 ± 0.05	0.50 ± 0.21	0.59 ^cd^ ± 0.28
B12	0.47 ± 0.13	1.20 ± 0.03	1.15 ± 0.20	0.71 ± 0.05	1.30 ± 0.44	0.86 ± 0.44	0.84 ± 0.14	1.10 ± 0.35	0.75 ± 0.73	1.05 ± 0.07	0.80 ± 0.47	0.92 ^e^ ± 0.37
B13	0.08 ± 0.03	0.35 ± 0.21	0.39 ± 0.15	0.40 ± 0.38	0.14 ± 0.11	0.25 ± 0.18	0.11 ± 0.07	0.26 ± 0.23	0.30 ± 0.08	0.16 ± 0.00	0.03 ± 0.02	0.23 ^a^ ± 0.20
B14	0.46 ± 0.31	0.49 ± 0.22	0.89 ± 0.28	0.56 ± 0.09	1.07 ± 0.68	0.52 ± 0.20	0.48 ± 0.11	0.57 ± 0.09	0.33 ± 0.12	0.37 ± 0.15	0.16 ± 0.00	0.55 ^bcd^ ± 0.32
Average	0.29 ^a^ ± 0.23	0.44 ^abc^ ± 0.27	0.57 ^c^ ± 0.29	0.52 ^bc^ ± 0.28	0.53 ^bc^ ± 0.30	0.36 ^abc^ ± 0.33	0.49 ^abc^ ± 0.29	0.34 ^ab^ ± 0.30	0.51 ^abc^ ± 0.36	0.32 ^ab^ ± 0.30	0.40 ^abc^ ± 0.31	

In the averages, values with different superscripts of lowercase letter in the same row or in the same column are significantly different (*p* < 0.01).

**Table 3:** Mean of the end-point pH values ± relevant standard deviation measured in co-cultivation with each LAB strain (from B1 to B14) crossed with each yeast strain (from L1 to L11) in reconstituted bovine sweet whey.

	L1	L2	L3	L4	L5	L6	L7	L8	L9	L10	L11	Average
B1	4.86 ± 0.06	4.50 ± 0.09	4.78 ± 0.03	4.76 ± 0.06	4.77 ± 0.04	5.15 ± 0.85	4.67 ± 0.02	5.77 ± 0.79	4.37 ± 0.04	5.42 ± 0.07	4.60 ± 0.04	4.87 ^e^ ± 0.50
B2	4.56 ± 0.21	4.30 ± 0.02	4.56 ± 0.08	4.42 ± 0.08	4.54 ± 0.23	5.10 ± 1.01	4.29 ± 0.03	5.59 ± 1.00	4.15 ± 0.03	4.98 ± 0.21	4.30 ± 0.06	4.62 ^cde^ ± 0.57
B3	4.59 ± 0.19	4.06 ± 0.09	4.73 ± 0.17	4.58 ± 0.18	4.61 ± 0.17	5.78 ± 0.30	4.35 ± 0.07	5.78 ± 0.34	3.95 ± 0.05	4.71 ± 0.23	4.05 ± 0.06	4.65 ^cde^ ± 0.62
B4	4.69 ± 0.05	4.15 ± 0.04	4.62 ± 0.22	4.56 ± 0.16	4.39 ± 0.06	5.65 ± 0.10	4.19 ± 0.06	6.04 ± 0.47	4.01 ± 0.04	4.75 ± 0.29	4.30 ± 0.06	4.66 ^cde^ ± 0.63
B5	4.87 ± 0.02	4.63 ± 0.20	4.65 ± 0.10	4.81 ± 0.03	4.76 ± 0.03	5.26 ± 0.76	4.63 ± 0.03	5.20 ± 0.34	4.75 ± 0.11	4.80 ± 0.31	4.31 ± 0.04	4.79 ^de^ ± 0.34
B6	4.79 ± 0.13	4.33 ± 0.18	4.55 ± 0.05	4.78 ± 0.13	4.76 ± 0.09	4.92 ± 0.24	4.43 ± 0.38	5.87 ± 0.88	3.97 ± 0.05	4.64 ± 0.18	4.18 ± 0.17	4.66 ^cde^ ± 0.55
B7	5.00 ± 0.17	4.57 ± 0.21	4.69 ± 0.06	4.87 ± 0.04	4.76 ± 0.03	5.90 ± 0.25	4.69 ± 0.02	4.79 ± 0.48	4.65 ± 0.33	4.25 ± 0.18	3.97 ± 0.09	4.74 ^cde^ ± 0.50
B8	5.52 ± 0.07	4.21 ± 0.11	4.44 ± 0.06	4.42 ± 0.27	4.29 ± 0.19	4.37 ± 0.03	4.20 ± 0.25	4.33 ± 0.07	4.09 ± 0.04	4.26 ± 0.07	4.22 ± 0.11	4.40 ^bcd^ ± 0.39
B9	5.77 ± 0.13	4.33 ± 0.14	4.60 ± 0.07	4.59 ± 0.12	4.61 ± 0.35	4.98 ± 0.10	4.37 ± 0.08	4.99 ± 0.26	3.95 ± 0.03	4.88 ± 0.03	4.72 ± 0.18	4.71 ^cde^ ± 0.47
B10	4.75 ± 0.24	4.10 ± 0.04	4.26 ± 0.05	4.30 ± 0.13	4.52 ± 0.31	4.46 ± 0.17	4.04 ± 0.08	4.36 ± 0.31	3.97 ± 0.09	4.60 ± 0.09	4.51 ± 0.07	4.35 ^abc^ ± 0.28
B11	5.17 ± 0.57	4.11 ± 0.06	4.22 ± 0.02	4.28 ± 0.12	4.42 ± 0.23	4.62 ± 0.15	4.00 ± 0.04	4.55 ± 0.18	4.01 ± 0.05	4.44 ± 0.10	4.28 ± 0.15	4.37 ^abc^ ± 0.37
B12	4.70 ± 0.40	3.90 ± 0.04	3.98 ± 0.03	4.04 ± 0.22	3.97 ± 0.19	4.08 ± 0.10	3.89 ± 0.08	4.02 ± 0.06	3.87 ± 0.07	4.15 ± 0.04	4.08 ± 0.18	4.06 ^ab^ ± 0.26
B13	5.39 ± 0.05	4.48 ± 0.06	4.51 ± 0.03	4.58 ± 0.09	4.90 ± 0.68	5.47 ± 0.40	4.53 ± 0.03	5.67 ± 0.15	4.16 ± 0.02	5.36 ± 0.12	5.00 ± 0.23	4.91 ^e^ ± 0.53
B14	5.07 ± 0.78	4.17 ± 0.58	3.93 ± 0.11	4.26 ± 0.91	3.76 ± 0.10	3.79 ± 0.08	3.73 ± 0.02	3.77 ± 0.04	3.89 ± 0.19	3.79 ± 0.03	3.81 ± 0.07	4.00 ^a^ ± 0.51
Average	4.97 ^de^ ± 0.44	4.27 ^ab^ ± 0.26	4.47 ^bc^ ± 0.27	4.52 ^bc^ ± 0.33	4.50 ^bc^ ± 0.38	4.97 ^de^ ± 0.72	4.29 ^ab^ ± 0.31	5.05 ^e^ ± 0.84	4.18 ^a^ ± 0.42	4.65 ^cd^ ± 0.46	4.31 ^ab^ ± 0.32	

In the averages, values with different superscripts of lowercase letter in the same row or in the same column are significantly different (*p* < 0.01).

## Data Availability

Data is contained within the article and Supplementary Materials.

## Supplementary Materials

The following supporting information can be downloaded at https://www.mdpi.com/article/10.3390/foods11101417/s1: Table S1: Composition of organic sunflower press cake from cold pressing used for the experimental trials, Table S2: Assimilation profiles of carbohydrates of the tested strains, Table S3: Starting (0 h) and final (48 h) Log CFU/g counts ± standard deviation of investigated microbiological groups in microcosms. In the first column, the first part of the label corresponds to the code of the bacterial strain, while the second part to the code of the yeast strain, Figure S1: image of a sample of the blend whey/sunflower press cake inoculated with micro-organisms (microcosm) poured in a 90 mm diameter Petri dish, Figure S2: Growth curves of the paired microbial associations in bovine sweet whey. The first part of the label corresponds to the code of the bacterial strain, while the second part to the code of the yeast strain. The incubation was carried out at 26 °C for 48 h.
