# Supplementary material for: Valorisation of Bovine Sweet Whey and Sunflower Press Cake Blend through Controlled Fermentation as Platform for Innovative Food Materials
Source: Foods. 2022 May 13;11(10):1417. doi: 10.3390/foods11101417 (PMC9142124; doi:10.3390/foods11101417)
Supplement: Supplementary file 1 [file foods-11-01417-s001.zip › foods-1703321-supplementary.pdf]

foods

MDPI

Article

Valorisation of Bovine Sweet Whey and Sunflower Press Cake Blend through Controlled Fermentation as Platform for Innovative Food Materials

Nicola Mangieri, Davide Ambrosini, Stefano Baroffio, Ileana Vigentini, Roberto Foschino * and Ivano De Noni

Department of Food, Environmental and Nutritional Sciences, Università degli Studi di Milano, 20122 Milano, Italy; nicola.mangieri@unimi.it (N.M.); dvd.ambrosini@gmail.com (D.A.); stefano.baroffio@studenti.unimi.it (S.B.); ileana.vigentini@unimi.it (I.V.); ivano.denoni@unimi.it (I.D.N.)
* Correspondence: roberto.foschino@unimi.it; Tel.: +39-02-58319166

Table S1. Composition of organic sunflower press cake from cold pressing used for the experimental trials (presented data were kindly provided by prof. Harald Rohm, Doris Jaros, Sophie Morejón Caraballo, Susanne Struck, from Technische Universität Dresden.

**Company**	**Ölmühle Moog GmbH, Klappendorf 1 01623 Lommatzsch, Germany**
Moisture	9.23%
Protein	19.53%
Fat	15.82%
Ash	4.91%
Dietary Fiber	51.42%
Particle size	< 1000 μm

Table S2. Assimilation profiles of carbohydrates of the tested strains.

Code	species	GAL	ACT	SAC	NAG	LAT	ARA	CEL	RAF	MAL	TRE	2KG	MDG	SOR	XYL	RIB	GLY	RHA	PLE	ERY	MEL	GRT	MLZ	GNT	LVT	MAN	LAC	INO	GLU	SBE	GLN	ESC
B1	*L. paracasei*	+	+	-	+	+	+	-	+	+	+	+	-	+	-	+	+	+	+	+	-	-	+	+	-	+	+	+	+	+	+	+
B2	*L. paracasei*	+	+	-	+	-	-	-	-	-	+	-	-	+	-	-	-	-	-	-	-	-	+	+	-	+	+	+	+	+	+	+
B3	*L. paracasei*	+	-	-	-	-	-	-	-	-	-	-	-	+	-	-	-	-	-	-	-	-	-	-	-	-	-	-	-	+	+	+
B4	*L. casei*	+	+	+	+	-	-	-	-	+	+	+	-	+	+	+	-	-	+	-	+	-	+	+	-	+	-	-	+	+	-	+
B6	*L. parakefiri*	+	-	-	-	-	-	-	-	-	-	-	-	+	+	-	-	-	-	-	-	-	-	-	-	-	-	-	-	+	+	-
B7	*L. kefiri*	+	-	+	+	-	-	-	-	-	-	-	-	-	+	+	-	-	-	-	-	+	+	+	+	-	-	+	-	+	+	-
B8	*L. paracasei*	+	+	+	+	-	-	-	-	+	+	+	-	+	+	+	-	-	+	-	+	-	+	+	-	+	-	-	+	+	-	+
B9	*L. paracasei*	+	+	+	+	-	-	-	-	-	+	+	-	+	-	+	+	+	+	+	+	-	+	+	+	-	+	+	+	-	+	+
B10	*L. lactis*	+	+	+	+	+	-	+	+	+	-	-	-	+	+	+	+	-	-	+	-	-	-	+	-	+	+	+	+	+	+	+
B11	*L. lactis*	+	+	+	+	-	-	+	+	+	+	+	+	+	+	+	+	+	+	+	+	+	-	-	+	+	+	-	+	+	+	-
B12	*L. lactis*	+	+	+	+	+	+	+	+	+	+	-	-	+	+	+	+	+	+	+	+	+	-	-	-	+	+	-	+	-	+	+
B13	*L. citreum*	+	+	+	+	+	+	+	-	+	+	+	+	+	+	+	+	+	+	+	-	-	+	+	+	+	+	+	+	-	+	-
B14	*L. pseudomes.*	-	+	-	-	-	-	-	-	-	-	-	-	-	-	-	-	+	-	+	-	-	-	-	-	-	-	-	+	-	+	-
L1	*D. hansenii*	+	-	+	+	+	+	+	+	+	+	+	+	+	-	-	+	+	+	-	-	-	+	-	-	+	+	-	+	+	-	+
L2	*K. lactis*	+	+	+	-	+	-	-	+	+	-	-	-	+	-	-	+	-	+	-	-	-	+	-	-	+	+	-	+	-	-	-
L3	*K. marxianus*	+	+	+	-	+	-	-	+	-	-	-	-	+	-	-	-	-	-	-	-	-	-	-	-	+	+	-	+	-	-	+
L4	*K.marxianus*	+	+	+	-	+	-	-	+	-	-	-	-	-	-	-	-	-	-	-	-	-	-	-	-	-	+	-	+	-	-	-
L5	*T. delbruecki*	+	-	+	-	+	-	-	+	-	-	+	-	-	-	-	+	-	-	-	-	-	-	-	-	-	-	-	+	-	-	-
L6	*P.fermentans*	-	-	-	+	+	-	-	-	-	-	-	-	-	-	-	-	-	-	-	-	-	-	+	-	+	-	-	+	+	+	-
L7	*K.marxianus*	+	+	+	+	+	-	+	+	-	-	-	+	-	+	-	+	-	-	-	-	-	-	-	-	+	+	-	+	-	-	-
L8	*P.fermentans*	-	-	+	+	+	-	-	-	-	-	-	-	-	-	+	-	-	-	-	-	-	-	+	-	-	-	-	+	-	+	-
L9	*K. lactis*	+	+	+	+	+	-	-	+	+	+	-	+	+	+	+	+	-	+	-	-	-	+	-	-	+	+	-	+	-	-	-
L10	*P. kluyveri*	-	-	-	-	-	-	-	-	-	-	-	-	-	-	-	+	-	-	-	-	-	-	+	-	-	-	-	+	-	+	-
L11	*S. cerevisiae*	-	-	+	-	-	-	-	-	+	-	-	-	-	-	-	+	-	-	-	-	-	-	+	-	-	-	-	+	-	+	-

[1] GAL: D-GALactose, ACT: cycloheximide (ACTidione), SAC: D-SACcharose (sucrose), NAG: N-Acetyl-Glucosamine, LAT: LacTic acid, ARA: L-ARAbinose, CEL: D-CELlobiose, RAF: D-RAFfinose, MAL: D-MALtose, TRE: D-TREhalose, 2KG: potassium 2-KetoGluconate, MDG: Methyl-αD-Glucopyranoside, SOR: D-SORbitol, XYL: D-XYLose, RIB: D-RIBose, GLY: GLYcerol, RHA: L-RHAmnose, PLE: PaLatinosE, ERY: ERYthritol, MEL: D-MELibiose, GRT: sodium GlucuRonaTe, MLZ: D-MeLeZitose, GNT: potassium GlucoNaTe, LVT: levulinic acid, MAN: D-MANnitol, LAC: D-LACtose, INO: INOsitol, GLU: GLUcose, SBE: L-SorBosE, GLN: GLucosamiNe, ESC: ESCulin ferric citrate.

Table S3. Starting (0h) and final (48h) Log CFU/g counts ± standard deviation of investigated microbiological groups (Lactic Acid Bacteria, Yeasts and Bacterial contaminants) in microcosms. In the first column, the first part of the label corresponds to the code of the bacterial strain, while the second part to the code of the yeast strain.

Sample	LAB t0	LAB t48	Yeasts t0	Yeasts t48	Bac. contaminants t0	Bac. contaminants t48
**B2 L2**	5.94 ± 0.66	8.97 ± 0.19	4.82 ± 0.51	7.43 ± 0.77	4.34 ± 0.21	5.59 ± 1.26
**B2 L5**	6.15 ± 0.63	9.03 ± 0.20	5.54 ± 0.39	6.91 ± 0.69	4.31 ± 0.20	5.45 ± 0.43
**B2 L7**	6.08 ± 0.59	9.07 ± 0.09	5.08 ± 0.33	7.44 ± 0.13	4.19 ± 0.16	5.05 ± 0.86
**B4 L2**	6.11 ± 0.66	9.18 ± 0.19	4.81 ± 0.51	5.78 ± 0.77	4.10 ± 0.21	4.93 ± 1.26
**B4 L5**	6.19 ± 0.33	8.56 ± 1.23	5.03 ± 0.95	6.84 ± 0.13	4.39 ± 0.28	5.67 ± 0.64
**B4 L7**	6.04 ± 0.23	8.96 ± 0.06	4.94 ± 0.44	7.44 ± 0.15	4.12 ± 0.26	4.27 ± 0.49
**B6 L2**	5.87 ± 0.79	7.97 ± 0.86	4.84 ± 0.47	6.96 ± 0.62	3.90 ± 0.55	4.68 ± 0.34
**B6 L5**	5.84 ± 1.10	8.62 ± 0.33	5.22 ± 0.33	7.32 ± 0.16	4.12 ± 0.22	4.95 ± 0.98
**B6 L7**	5.37 ± 0.41	8.67 ± 0.15	5.34 ± 0.31	7.70 ± 0.32	4.03 ± 0.09	5.18 ± 0.50
**B8 L2**	5.99 ± 0.79	9.18 ± 0.27	4.44 ± 0.80	7.15 ± 0.55	3.88 ± 0.43	3.76 ± 1.06
**B8 L5**	6.30 ± 0.32	9.48 ± 0.16	5.71 ± 0.24	6.39 ± 0.68	3.84 ± 0.03	4.54 ± 1.39
**B8 L7**	6.33 ± 0.57	9.02 ± 0.23	5.24 ± 0.43	7.36 ± 0.22	3.90 ± 0.20	4.43 ± 0.51
**B12 L2**	6.32 ± 0.47	9.30 ± 0.24	4.49 ± 0.49	7.21 ± 0.30	4.07 ± 0.06	3.20 ± 0.35
**B12 L5**	6.87 ± 0.22	9.41 ± 0.15	5.55 ± 0.26	6.49 ± 0.07	4.20 ± 0.17	3.05 ± 0.11
**B12 L7**	6.48 ± 0.40	9.39 ± 0.19	5.04 ± 0.43	7.04 ± 0.36	4.10 ± 0.37	3.79 ± 0.77
**B14 L2**	5.93 ± 0.75	8.24 ± 0.72	4.70 ± 0.62	7.20 ± 0.36	3.67 ± 0.52	5.20 ± 0.67
**B14 L5**	5.50 ± 1.14	8.62 ± 0.86	5.24 ± 0.68	6.96 ± 0.78	4.01 ± 0.16	5.00 ± 0.38
**B14 L7**	6.49 ± 0.24	8.59 ± 0.21	5.37 ± 0.38	7.47 ± 0.23	4.00 ± 0.05	4.36 ± 1.28

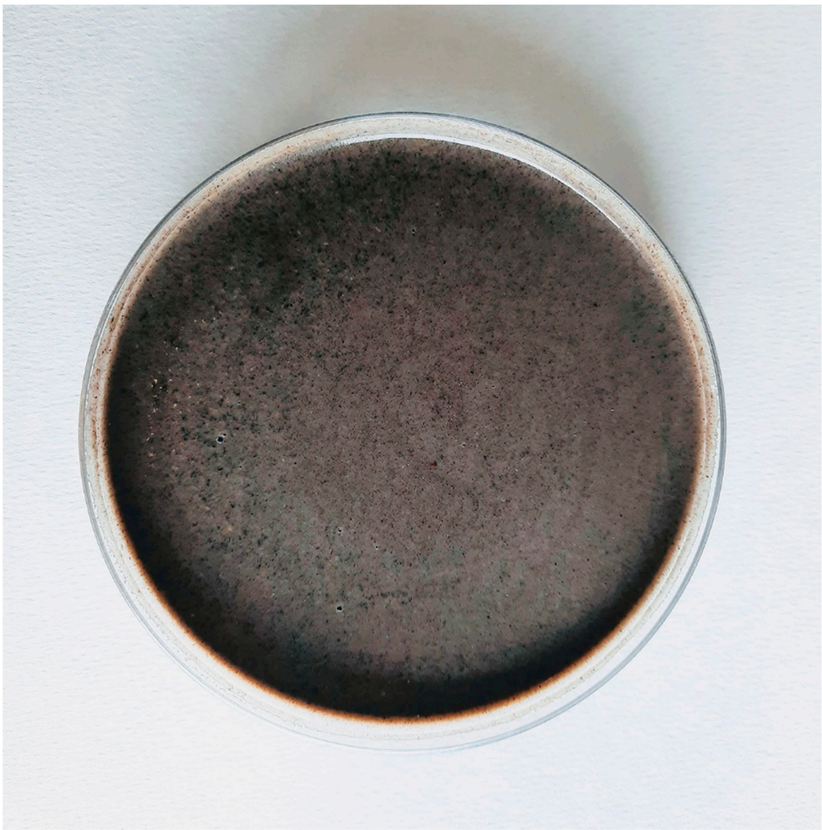

Figure S1. Image of a sample of the blend whey/sunflower press cake inoculated with micro-organisms (microcosm) poured in a 90 mm diameter Petri dish.

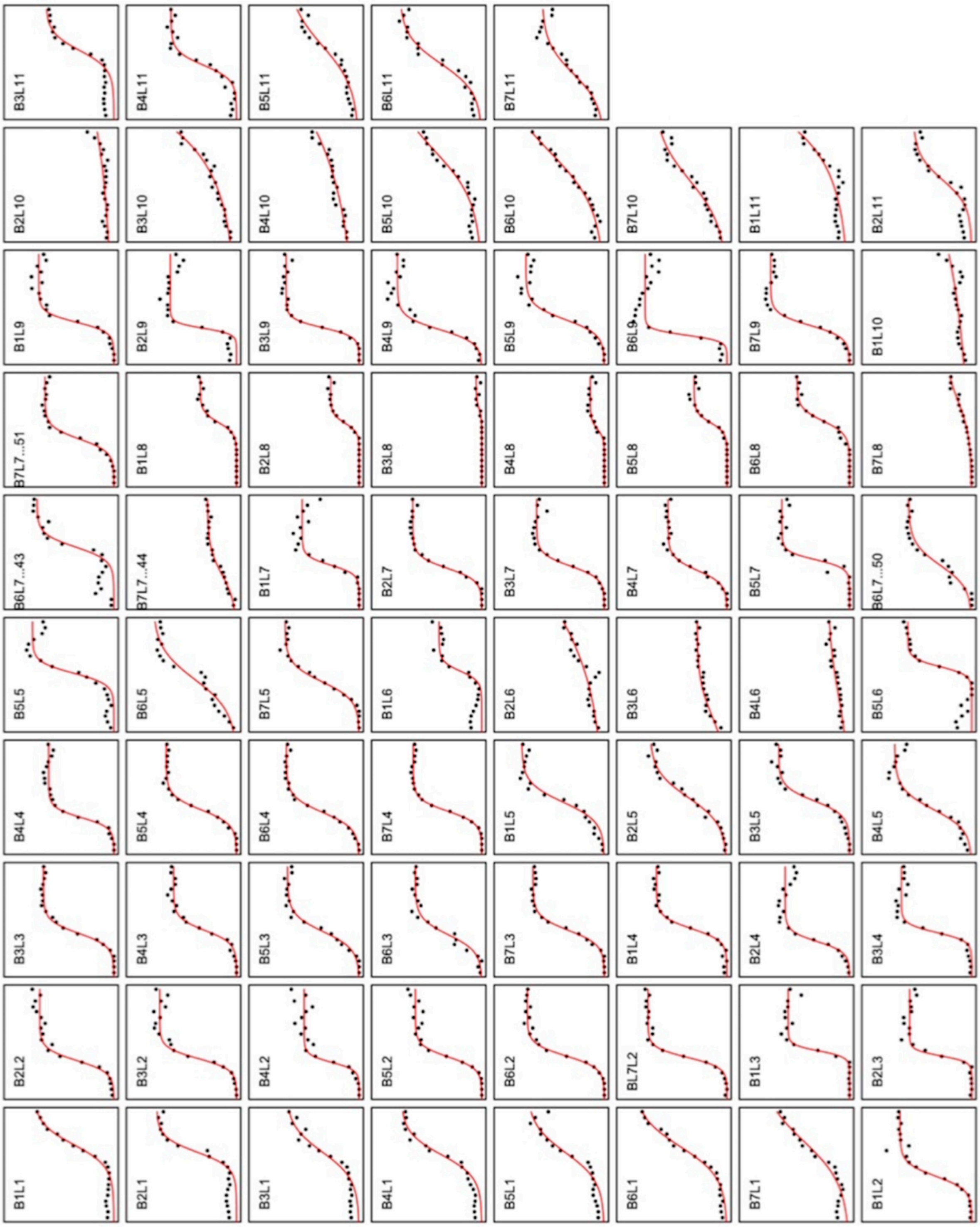

Figure S2. Growth curves of the paired microbial associations in bovine sweet whey. The first part of the label corresponds to the code of the bacterial strain, while the second part to the code of the yeast strain. The incubation was carried out at 26 °C for 48 h.
